# Medical screening tests and vaccination among hospital-based physicians in Israel

**DOI:** 10.1186/s12913-022-08714-8

**Published:** 2022-11-10

**Authors:** Sameeh Eltalakat, Berjas Abu Gariba, Roni Peleg, Daniel Kaplan, Yulia Treister-Gotzman

**Affiliations:** 1 Department of Family Medicine and Siaal Research Center for Family Practice and Primary Care, Faculty of Health Sciences, Ben-Gurion University of the Negev, POB 653, 84105 Beer-Sheva, Israel; 2 Clalit Health Services, Southern District, Tel Aviv, Israel; 3 Ear, Nose, and Throat, and Head and Neck Surgery Ward, Soroka Medical Center, Beer-Sheva, Israel

**Keywords:** Preventive medicine, Immunization, Screening tests, Physicians, Mammography, Occult blood test

## Abstract

**Background:**

Very few studies have evaluated physician participation in screening tests and vaccinations. The aim was to evaluate attitudes and actual practice of screening tests and vaccinations among hospital-based physicians and to identify factors that predict actual performance.

**Methods:**

A cross-sectional study including 220 physicians in various specializations. The study was conducted between January 1, 2017 and December 10, 2017. The statistical analysis was performed during October, 2020 and completed in October 25, 2020.

**Results:**

The vast majority of physicians (94%) believed in the importance of screening tests for physicians, but less than half actually underwent the tests themselves. A high percentage of physicians measured their BMI (84.5%) and had a serum lipid profile test (67.7%) and complete blood counts (75%) over the previous five years, but less than a third of female physicians over 50 years of age had a mammogram and only 8% of the physicians over 50 had a fecal occult blood test. A high percentage of physicians were vaccinated for Hepatitis A and B, chickenpox and measles (66-96% for the various vaccinations), but only 41% had an influenza vaccination in the previous year. In a multivariate logistic regression model, physicians who believed that hospital physicians bore a responsibility for recommending screening tests to their patients were more likely to have their BMI and their blood pressure checked (OR = 2.234, *P* = 0.007). They were more likely to undergo laboratory testing (OR = 4.082, *P* < 0.0001) and tended to get vaccinated more (OR = 1.907, *P* = 0.051).

**Conclusion:**

The rate of screening tests and vaccinations among hospital-based physicians is sub-optimal and very low for fecal occult blood testing and mammograms. Structured programs are required to implement screening tests and vaccinations among physicians.

## Background

Primary and secondary prevention are an important element of public health, usually with a higher cost/benefit ratio than the treatment of symptomatic diseases or their complications [1]. There is a problem in implementing primary and secondary prevention programs because the target population is usually composed of healthy individuals. It can be challenging to convince such people of the need for preventive medicine. Physicians have an important, central position in educating the general population on a healthy lifestyle and on screening tests that have been shown to prevent mortality. Physicians should also practice preventive measures personally.

A literature review showed that physicians usually relate to their own health only when they have serious diseases [2]. They work when sick [3], and treat themselves either on their own or in an informal process through corridor consultations alongside formal care [3, 4]. A study of a large cohort of physicians in the United States showed that more than one third of the physicians did not participate in routine checkups, did not undergo screening tests, and did not get vaccinated [5]. There is research evidence that patients’ decisions to undergo screening tests is strongly affected by their primary care physician’s recommendation [6, 7]. Physicians who have a healthier lifestyle and who believe in the importance of preventive medicine were more positive about advising their patients on health promotion [7, 8].

Preventive medicine guidelines in Israel are based on the recommendations of a task force on health promotion and disease prevention [9]. The guidelines were updated by a committee that was composed of representatives of different medical specialties and convened in 2013. It is important to note that Israel has a national health law that covers subsidized medical services for all residents of Israel. Early detection screening tests and vaccinations are provided to the entire population free of charge. The implementation of activities related to health promotion and disease prevention is an important part of the work of family physicians in Israel and recommendations by hospital specialists are also of great importance. The results of a study on the conduct of screening tests among family physicians in Israel, a sector that bears great responsibility for the implementation of screening tests in the general Israeli population, showed that less than one third of family physicians in Israel underwent screening tests themselves even though more than 50% believed that screening tests are efficacious [10].

The aim of the present study was to evaluate attitudes towards and the practice of screening tests and vaccinations among hospital physicians in southern Israel. A second aim was to identify the characteristics of these physicians that predict whether they do or do not implement these actions themselves.

## Methods

The study was conducted in the Soroka University Medical Center in the city of Beer-Sheva, Israel. It is the central hospital for the Negev region of Israel and the fourth largest hospital in Israel. It provides medical services for one million residents of southern Israel, primarily secondary and tertiary care. The study was conducted between January 1, 2017 and December 10, 2017.

The study instrument was a self-administered questionnaire that included socio-demographic items, questions to assess attitudes and questions on the actual practice of screening tests and vaccinations and reasons for not practicing them. The questionnaire was adopted from a previous study that was carried out in southern Israel [10]. That study, as mentioned, addressed attitudes and practice related to screening and vaccinations among primary care physicians. For that study the questionnaire was formulated after a comprehensive review of the literature, face validation, a pilot study, and revision in light of the pilot study results [10]. Screening tests recommendations, that were addressed in the questionnaire, are part of the preventive medicine guidelines in Israel and are based on the recommendations of a task force on health promotion and disease prevention [9] The guidelines are based on age group and provide a detailed description of recommended actions. These recommendations were developed and are updated at in accordance with the recommendations of the American, Canadian, and European task forces, and the recommendations of the Israel Ministry of Health, considering epidemiological data and the unique structure of the healthcare system in Israel. Most of the recommendations meet the criteria for Grade A and B recommendations of the US Task Force (USPTF). Several guidelines were included even though they are based on expert opinion alone without strong research evidence at this time. It is important to note that that the test of choice for the early detection of colorectal cancer, which was affirmed as a nationwide program, is annual fecal occult blood testing using a high-sensitivity kit for individuals aged 50–75 years, and a colonoscopy for those with a positive fecal occult blood test. Colonoscopy is the test of choice in high-risk individuals only, but other individuals can choose to undergo elective colonoscopy in a private medicine setting. Doing a baseline ECG tracing for future comparison purposes is part of the preventive medicine recommendations, but this subject is controversial.

The questionnaire was distributed to physicians in the framework of staff activities for residents and specialists in various hospital departments. The statistical analysis was performed during October 2020 and completed on October 25, 2020.

Categorical variables such as sex, country of birth, and adherence to screening tests are presented as frequencies and percentages. Continuous variables such as age are described as means and standard deviations. Categorical variables were tested for differences by the chi-square test or Fisher’s exact test in accordance with the size of the cells. Differences for continuous variables were identified by one-way ANOVA. A multivariate logistic regression model was built for the association of independent factors with: (a) implementation of anthropometric tests such as blood pressure over the previous year and BMI over the last five years, (b) implementation of blood tests and lipidograms over the last five years, and (c) and getting recommended vaccinations.

The sample size was calculated based on the assumption that the rate of screening tests and vaccination among hospital physicians is about 30 ± 5%. At the level of statistical significance 5%, at least 165 physicians would have to be interviewed.

All methods were performed in accordance with the relevant guidelines and regulations. The ethics committee of Soroka University Medical Center approved the study (approval #0074-16-SOR). Informed consent was waived by the same ethics committee that approved the study: The ethics committee of Soroka University Medical Center.

## Results

### Characteristics of the study population

The study population consisted of 220 physicians including 113 women (51.4%). The majority of the participants (74.5%) were born in Israel and the mean age was 41.8 ± 9.3 years. The majority were board-certified (54.5%) and the others were residents and interns. Most of the physicians (85.5%) were healthy without chronic diseases that necessitated chronic drug treatment. The basic characteristics of the study population are presented in Table 1.

**Table 1:** Characteristics of the study population

Variable	Value
**Family status** [N (%)]	
Single	50 (22.8)
Married	136 (62.1)
Divorced	31 (14.2)
Widowed	2 (0.9) missing = 1
**Sex** [N (%)]	
Male	107 (48.6)
Female	113 (51.4)
**Age**	
Mean ± SD	41.8 ± 9.3
Median	41.5
Range	22–68
**Country of birth** [N (%)]	
Israel	164 (74.5)
Other	56 (25.5)
**Years in Israel (for born abroad)**	
Mean ± SD	16.6 ± 8.8
Median	16
Range	3–54
**Professional status [N (%)]**	
Specialist	120 (54.5)
Resident	86 (39.1)
Intern	14 (6.4)
**Country of medical studies [N (%)]**	
Israel	90 (40.9)
Other	130 (59.1)
**Chronic disease requiring chronic drug treatment [N (%)]**	
Yes	32 (14.5)
No	188 (85.5)

### Attitudes towards screening tests

Most of the physicians believed in screening tests with 125 (56.8%) agreeing to a great degree and 37.3% agreeing with some qualifications. Similar results were seen in the attitudes of the physicians towards screening tests for their family members (45.9% and 46.6%, respectively). Over 92% of the physicians believed that the responsibility for screening tests by patients rested with the family physicians, and 60% believed that hospital-based physicians should also take responsibility for this. The attitudes of physicians towards screening tests are presented in Table 2.

**Table 2:** Attitude towards screening tests

Variable	N (%)
**Should doctors undergo screening tests?**	
Yes, definitely	125 (56.8)
Yes, with qualifications	82 (37.3)
Not so much	13 (5.9)
Not at all	0
**Should your family members undergo screening tests?**	
Yes, definitely	101 (45.9)
Yes, with qualifications	102 (46.4)
Not so much	17 (7.7)
Not at all	0
**Should the responsibility for recommending screening tests to patients be on family doctors?**	
Yes, definitely	142 (64.8)
Yes, with qualifications	60 (27.4)
Not so much	17 (7.8)
Not at all	0
**Should the responsibility for recommending screening tests to patients be on hospital consultants?**	
Yes, definitely	29 (13.2)
Yes, with qualifications	103 (46.8)
Not so much	79 (35.9)
Not at all	9 (4.1)

### Actual implementation of screening tests

In general, less than half of the physicians reported that they actually underwent general screening tests, with more than half citing lack of time as the reason for not. 161 participants (84.5%) calculated their BMI, 165 (75%) had a complete blood count, and 149 (67.7%) had a lipidogram over the previous five years. Only half had their blood pressure measured and only 60% of the women had a gynecological examination over the previous year. Only 40% got a flu vaccination in the previous year with some not remembering if they got one or not. In contrast, the rate for other vaccinations including HAV (93.6%), HBV (95.5%), tetanus (71.8%), rubella (70%) and measles (66.8%) were relatively high. Among physicians aged 40–64 years 62.7% had an eye examination over the previous five years and only 48.3% had a skin examination. Similarly, only 9% of the participants over the age of 50 years had a fecal occult blood test over the previous year and only one third of women physicians over the age of 50 underwent mammography in the previous two years. Details on the actual practice of screening test among the hospital physicians are presented in Table 3.

**Table 3:** Actual practice of screenings tests

Variable	N (%)
**Do you undergo screening tests on a regular basis?**	
Yes	125 (56.8)
No	82 (37.3)
Not relevant	13 (5.9)
**If not, what is the reason**?	
I don’t believe in it	6 (7.3)
I don’t have the time for it	43 (52.4)
I will do it as soon as possible	17 (20.7)
I don’t always remember to do it	15 (18.3)
Other	1 (1.2) missing = 2
**Has your BMI been calculated over the last 5 years?**	
Yes	186 (84.5)
No	34 (15.5)
**When did you last have your blood pressure checked?**	
In the last year	109 (49.5)
1–2 years ago	32 (14.5)
2–5 years ago	8 (3.6)
More than 5 years ago	2 (0.9)
Never	4 (1.8)
I don’t remember	65 (29.5)
**Did you have a lipidogram over the last 5 years?**	
Yes	149 (67.7)
No	71 (32.3)
**Did you have a complete blood count over the last 5 years?**	
Yes	165 (75.0)
No	55 (25.0)
**For women only – did you have a gynecologic examination in the last year?**	
Yes	64 (60.4)
No	33 (31.1)
I don’t remember	9 (8.5) missing = 7
**For ages 40–64 only – did you have your vision checked over the last 5 years?**	
Yes	74 (62.7)
No	42 (35.6)
I don’t remember	2 (1.7) missing = 2
**For ages 40–64 only – were you examined by a dermatologist over the last 5 years?**	
Yes	57 (48.3)
No	60 (50.8)
I don’t remember	1 (0.8) missing = 2
**For ages 40–64 only – did you have a baseline ECG tracing?**	
Yes	41 (34.7)
No	75 (63.6)
I don’t remember	2 (1.7) missing = 2
**For ages over 50 years only – did you have a fecal occult test in the last 5 years?**	
Yes	10 (8.5)
No	104 (88.1)
I don’t remember	4 (3.4) missing = 2
**For women over 50 years only – did you have a mammography over the last 2 years?**	
Yes	5 (31.3)
No	9 (56.3)
I don’t remember	2 (12.5) missing = 2
**For women between 40–49 years – did you have a breast exam by your family physician?**	
Yes	6 (7.8)
No	62 (80.5)
I don’t remember	9 (11.7) missing = 8
**Did you get a flu vaccination in the last year?**	
Yes	90 (40.9)
No	105 (47.7)
I don’t remember	25 (11.4)
**HAV?**	
Yes	205 (93.6)
No	9 (4.1)
I don’t remember	5 (2.3)
**HBV?**	
Yes	210 (95.5)
No	7 (3.2)
I don’t remember	3 (1.4)
**Diphtheria/tetanus over the last 10 years?**	
Yes	158 (71.8)
No	39 (17.7)
I don’t remember	23 (10.5)
**Chickenpox**?	
Yes	154 (70.0)
No	24 (10.9)
I don’t remember	42 (19.1)
**Measles?**	
Yes	147 (66.8)
No	24 (10.9)
I don’t remember	49 (22.3)

### Comparison between men and women, residents and specialists and age groups on attitudes and actual practice

There were no significant differences among the subgroups by sex, resident/specialist status, or age group on attitudes toward and practice of screening tests.

### Multivariate logistic regression model for the prediction of actual practice of screening tests among the physicians

The models are presented in Table 4. Physicians who suffered from chronic diseases that required chronic drug treatment (OR = 2.54, *P* = 0.038) and those who believed that hospital physicians bore a responsibility for recommending screening tests to their patients (OR = 2.23, *P* = 0.007) were more likely to have their BMI measured over the previous five years and their blood pressure checked in the previous year. Physicians who believed that hospital physicians also bore a responsibility for recommending screening tests to their patients were more likely to undergo laboratory testing (OR = 4.08, *P* < 0.0001) and tended to get vaccinated more than other physicians in the hospital (OR = 1.907, *P* = 0.051).

**Table 4:** Multivariable regression models for screening tests and vaccination practice among hospital physicians

	OR	95% CI	P
**Model 1**			
** Had a complete blood count and a lipidogram over the last five years**			
Sex (Male)	1.023	0.544–1.923	0.944
Age (years)	1.014	0.976–1.053	0.474
Suffers from a chronic disease with chronic drug treatment	2.632	0.791–8.766	0.115
Believe in screening tests for physicians	0.921	0.257–3.293	0.899
Believe that the responsibility for screening tests for patients lies with hospital consultants	**4.082**	2.209–7.544	< **0.0001**
**Model 2**			
** Measured blood pressure in the last year and BMI over the last 5 years**			
Sex (Male)	1.130	0.634–2.015	0.679
Age (years)	0.998	0.964–1.032	0.891
Suffers from a chronic disease with chronic drug treatment	**2.539**	1.054–6.118	**0.038**
Believe in screening tests for physicians	1.053	0.326–3.402	0.931
Believe that the responsibility for screening tests for patients lies with hospital consultants	**2.234**	1.240–4.025	**0.007**
**Model 3**			
** Had all the recommended vaccinations**			
Sex (Male)	1.525	0.820–2.837	0.182
Age (years)	1.000	0.964–1.037	0.995
Suffers from chronic diseases with chronic drug treatment	1.709	0.699–4.182	0.24
Believe in screening tests for physicians	2.214	0.469–10.450	0.315
Believe that the responsibility for screening tests for patients lies with hospital consultants	**1.907**	0.998–3.645	**0.051**

## Discussion

Research to date on physicians’ health has focused on mental health, burnout and addiction. Much less research has focused on whether physicians actually practice screening tests and preventive medicine, which are an important aspect of public health. Physicians are known to disparage their own health in favor of personal and professional commitments [11]. Although most of the physicians believe in the efficacy of screening tests for themselves and their family, no more than 50% report that they actually underwent those tests. In a study similar to the present one that was conducted among family physicians several years ago there was a high percentage of physicians who believed in screening tests, but an even lower rate (less than one third) who actually underwent the tests [10]. The most common reason for not being tested among family physicians in the previous study, and among hospital physicians in the present study was lack of time. In a study conducted in Canada there was a much higher rate of physicians who underwent screening tests [12]. The percentage of physicians over the age of 50 who had a fecal occult blood test and a mammography was particularly low; much lower than that reported among patients [13]. The results of a study from Saudi Arabia showed an even lower rate of mammography exams among healthcare workers [14]. There was a higher rate of mammography exams among female gynecologists in the United States than among all hospital physicians, but the rate was still lower than among female patients [15]. In contrast to the study on family physicians in Israel, in the present study of hospital physicians there was no difference between older and younger or residents and specialists in their attitudes towards screening tests. It is of particular importance that physicians, who are exposed to various diseases in the course of their work, get the recommended vaccinations.

While the percentage of physicians getting vaccinations that are given once in a lifetime or once in several years was high, the rate for annual influenza vaccinations was about 40%. This rate was similar among family physicians in Israel [10]. A similar rate for influenza and other vaccines was found among pediatricians in Vienna [16]. A study from Italy showed an even lower rate for influenza vaccinations among healthcare workers at 14.5%, although the rate was higher among physicians compared to other healthcare workers [17]. In contrast, a higher percentage of physicians in Greece were vaccinated for seasonal flu (55%), but the rates for tetanus, hepatitis, and measles was much lower [18]. In Switzerland 15–30% of healthcare workers reported getting vaccinated [19]. While in the present study the main reason for not getting vaccinated was lack of time and some physicians said they didn’t remember if they did or did not get vaccinated, in other countries physicians did not believe in the importance of getting vaccinated themselves and were also concerned about adverse effects [18, 19]. While influenza vaccination is beneficial to the health of physicians and protects against work absenteeism in the year of the vaccination [17], they also prevent mass infection of patients [16], Although over 92% of hospital physicians believed that the responsibility for screening tests is on family physicians, only 60% believed that they also had responsibility for recommending screening tests to their patients. Interestingly, this attitude was significantly associated with BMI and blood pressure measurements as well as complete blood counts and borderline associated with all vaccinations in a regression model. Other studies have shown that physicians who practice health promotion and screening tests themselves are more likely to recommend these tests to their patients [15, 20]. Thus, the practice of health promotion by physicians themselves can also improve health promotions and the practice of screening tests among patients.

### Limitations

This study was based on interviews, so the well-known potential for recall bias in this type of study is the first limitation. It is possible that the small sample of 16 respondents that responded to the question on mammography (women aged 40–64) was not enough to provide an accurate estimate of rates of mammography among older female doctors in Israel. Statistical power calculations were based on the assumption that all questions would apply to all respondents, and did not take into account that some questions apply only to a small subset. In addition, there are missing data for several questions, but in no case was there more than 10% of missing answers for any item.

## Conclusion

The rate of screening tests and vaccination among hospital physicians is low and especially low for certain tests such as fecal occult blood testing and mammography. The rate of influenza vaccination is unsatisfactory. Much more attention is paid to the practice of preventive medicine in the general population, but physicians should not be any different in this respect. A multilevel approach is required to provide solutions for and improve preventive medicine implementation among physicians. At the individual level, the practice of self-care should be perceived as essential for the physician’s individual well-being and improved professional provision of patient care. This requires enhanced self-awareness and insight to prioritize self-care, identifying barriers to its implementation and ways to overcome them. At the system level, beginning with medical school education, the model of healthy physicians who take care of themselves despite the high pressures of the their profession, should be encouraged. Evidence should be provided that healthy physicians deliver better care to their patients. This problem should also be addressed by healthcare policy makers. Structured programs with protected time and coverage should be designed for the implementation of screening tests and vaccinations among physicians.

## Data Availability

The data that support the findings of this study is available on request from the corresponding author.
