# Correction: Medical screening tests and vaccination among hospital-based physicians in Israel

**DOI:** 10.1186/s12913-022-08906-2

**Published:** 2022-12-01

**Authors:** Sameeh Eltalakat, Berjas Abu Gariba, Roni Peleg, Daniel Kaplan, Yulia Treister-Goltzman

**Affiliations:** 1 Department of Family Medicine and Siaal Research Center for Family Practice and Primary Care, Faculty of Health Sciences, Ben-Gurion University of the Negev, POB 653, 84105 Beer-Sheva, Israel; 2 Clalit Health Services, Southern District, Tel Aviv, Israel; 3 Ear, Nose, and Throat, and Head and Neck Surgery Ward, Soroka Medical Center, Beer-Sheva, Israel

**Correction: BMC Health Serv Res 22, 1335 (2022)**

**https://doi.org/10.1186/s12913-022-08714-8**

Following publication of the original article [1], the authors identified an error in the author name of Yulia Treister-Goltzman.

The incorrect author name is: Yulia Treister-Gotzman

The correct author name is: Yulia Treister-Goltzman

The author group has been updated above and the original article [1] has been corrected.
